# The Th17/IL-23 Axis and Natural Immunity in Psoriatic Arthritis

**DOI:** 10.1155/2012/539683

**Published:** 2012-04-01

**Authors:** Shinji Maeda, Yoshihito Hayami, Taio Naniwa, Ryuzo Ueda

**Affiliations:** Department of Medical Oncology and Immunology, Nagoya City University Graduate School of Medical Sciences, Nagoya, Aichi 467-8601, Japan

## Abstract

Psoriatic arthritis (PsA) is a chronic inflammatory skin disease that causes enthesitis and destructive arthritis and significantly lowers patient quality of life. Recognition of the two target organs (the skin and joints) involved in the immunopathophysiology of PsA helped in elucidating the pathology of various systemic autoimmune diseases targeting multiple organs. Recent advances in immunology and genetics have made it clear that acquired immunity, especially that mediated by the Th17/IL-23 axis, plays an important role in the inflammatory pathology observed in psoriasis and PsA. Additionally, involvement of natural immunity has also been suggested. Microbial infection has been known to trigger psoriasis and PsA. Recent clinical studies using biopharmaceuticals, such as tumor-necrosis-factor- (TNF-) **α** inhibitors and IL-12/23 p40 antibodies, indicate that studies need not be based only on the immunological phenomena observed in PsA pathology since disease pathology can now be verified using human-based science. Considering this aspect, this paper discusses the immunopathology of PsA compared to psoriasis (cutaneous) and rheumatoid arthritis in humans and immunopathology of PsA with respect to the Th17/IL-23 axis and microbial infection.

## 1. Introduction

Psoriasis is a typical inflammatory keratosis, characterized by inflammation, telangiectasia, hyperproliferation, and abnormal differentiation of epidermal cells, and it is sometimes complicated by arthritis. There is considerable variation in reports concerning the transition from psoriasis to psoriatic arthritis (PsA), with figures ranging from 6% to 42%. The onset of cutaneous psoriatic eruptions usually precedes that of PsA; a study involving 1000 patients with PsA showed that cutaneous eruptions preceded the onset of PsA in 86% of cases by more than 12 years [1, 2]. PsA often causes destructive arthritis and arthropathy, which considerably decrease patient quality of life. Thus, clinicians must anticipate when patients with psoriasis vulgaris will progress to arthropathic psoriasis and intervene decisively in the early stages of onset of arthropathy, therapeutically targeting psoriatic skin and joint inflammation.

Psoriasis can affect two target organs—the skin and synovial membrane—and its main pathogenesis is considered to be chronic inflammation. The cause of this disease is probably based on the common foundations of autoimmune or autoinflammatory pathology. The mechanism by which this occurs begins with antigen-presenting cells, which are presumed to activate cutaneous and synovial membrane T cells, although the autoantigens important for this process remain unclear. These activated T cells then move from circulation to the target organs, and chronic inflammation occurs in the skin and synovial fluid. This process is considered to occur before the onset of clinical symptoms.

To date, studies have indicated several key differences between the inflammatory pathology of psoriasis and PsA. Because the pathology of PsA differs from that of cutaneous psoriatic lesions and at present it is difficult to obtain synovial samples for pathological analyses, elucidation of the pathology of associated arthropathy is lagging behind that of cutaneous eruptions. In patients with PsA, cutaneous eruptions and arthropathy do not necessarily exhibit the same disease characteristics, and their pathological relationship remains unclear.

Causes of psoriasis and PsA remain unclear, although new disease-susceptibility genes have recently been identified through genome analysis [3]. These discoveries have highlighted the importance of natural immunity, particularly in the immunopathology of T cells and cytokines, especially T helper (Th) 17 cells. In addition, the introduction of biopharmaceuticals for treatment of autoimmune diseases has contributed considerably to elucidation of pathology of rheumatism and psoriasis. Targeted therapy, focused on anticytokine preparations, has recently been introduced for treatment of various autoimmune diseases including rheumatoid arthritis (RA), and dramatic results are being obtained.

PsA is no exception. The use of biopharmaceuticals, such as tumor-necrosis-factor- (TNF-) *α* inhibitors, for treatment of psoriasis and PsA is leading to a paradigm shift [4–6]. Although the immunopathology of PsA, involving both cutaneous and synovial targets, is complex, genome analysis and targeted therapy with biopharmaceuticals have increased our understanding of the immunopathology of PsA.

This paper discusses the immunopathology of PsA by comparing the pathology of PsA with that of cutaneous psoriatic lesions and RA, a representative example of autoimmune arthritis, to provide a better understanding of this subject.

## 2. CD4+ T Cells in Psoriasis

T-cell suppression treatment for psoriasis in humans has shown that T cells play an important role in psoriatic pathology. T-cell activation inhibitors, including cyclosporine, IL-2 toxin, and Alefacept (lymphocyte function-associated antigen-3-Ig LFA-3-Ig), have been used to successfully treat psoriasis, indicating that T cells are intimately involved in the pathology of this disease.

An interesting study involved the transplantation of unaffected skin of a patient with psoriasis to SCID (severe combined immune deficiency) mice. The introduction of activated CD4+ T cells induced cutaneous psoriatic lesions, although the same did not occur with CD8+ T cells. This additionally clarified the significant role of CD4+ T cells in the onset of psoriasis [7].

Th1 cells have previously been considered to be significantly involved in psoriasis because of the large number of interferon-gamma- (IFN-*γ*-) producing cells observed in areas of cutaneous eruptions. However, the discovery of IL-17-producing helper T cells (Th17 cells) [8] made it clear that these were the cells most significantly involved in psoriasis [9–11].

## 3. The Role of the Th17/IL-23 Axis in Psoriasis

Th17 cells are a newly identified subset of helper T cells that produce interleukin- (IL-) 17A, IL-17F, IL-21, and IL-22 [12] and differ functionally from Th1 and Th2 cells. Analysis of IL-17-deficient mice [13] suggested that specific conditions previously considered to be suppressed by Th1 cells, including specific infections and autoimmune diseases (e.g., autoimmune arthritis [14], experimental autoimmune encephalomyelitis (EAE), and psoriasis [15]) may actually be inhibited by Th17 cells. Like Th1 and Th2 cells, Th17 cells appear to have evolved to induce acquired immune responses against microbes, such as bacteria. Abnormal Th17 responses are believed to play a significant role in the onset of various autoimmune diseases.

The Th17 cell lineage differs from that of Th1 and Th2 cells. Orphan Nuclear Receptor ROR*γ*t (retinoid-related orphan receptor gamma t) have been identified as specific Th17 transcription factors [16]. ROR*γ*t cooperates with signal transducer and activator of transcription 3 (STAT3) to induce production of IL-23 receptors, which play an important role in maintenance and amplification of Th17 cells. IL-23 receptors promote IL-17 transcription and Th17 cell differentiation via enforced ROR*γ*t expression.

IL-23 is a member of the IL-12 cytokine family, which forms a heterodimer comprising p40 and p19 subunits. It is produced by antigen-presenting cells, such as dendritic cells and macrophages.

IL-23 also binds to receptors to which the IL-12R*β*1 and IL-23R subunits have already been bound. IL-23 acts on cells that have differentiated into Th17 cells, potentiating ROR*γ*t activity, and participates in maintenance and proliferation of Th17 cells. Induction of Th17 cells alone by IL-6 and TGF-*β* results in production of IL-10, which inhibits inflammation. However, IL-23 causes inherent activation of Th17 effector cells [17, 18]. If IL-23 levels are low, then Th17 cells will be absent and IL-17 production will be scarce [19, 20]. Thus, IL-23 is indispensable for Th17 effector functions in immune disorders and maintenance of Th17 cells.

Numerous studies analyzing psoriatic cutaneous lesions revealed that the Th17/IL-23 axis is important in psoriasis pathogenesis. Th17 cells secrete IL-22 in addition to IL-17, with increased IL-17 and IL-22 levels seen in psoriatic areas [21–25]. These cells can also increase serum and cutaneous eruption site IL-23 expression in psoriatic patients [26], and increased number of Th17 and Th22 cells (CD4+ T-helper cell subset that produces IL-22, but not IFN-*γ* or IL-17) have also been observed [27]. Expression of the surface markers IL-23R and CC chemokine receptor (CCR) 6, considered to be involved in pathogenesis, is also seen with these Th17 cells. Th22 cells are believed to be especially involved in skin inflammation.

Cytokines, such as IL-22, IL-9, and IL-20, that are produced by Th17 and Th22 cells can activate STAT3 in epidermal keratinocytes and inhibit their differentiation, while promoting cell migration and proliferation [23, 28–30]. It has also been confirmed experimentally that these cytokines induce inflammatory chemokines and promote cutaneous acanthosis and parakeratosis [15, 24].

As mentioned previously, IL-23 is a cytokine that is necessary for the function and maintenance of Th17 effector cells. Dermal myeloid dendritic cells play an important role in activation of Th17 cells, which are involved in psoriatic lesion formation, through production of IL-23 and IL-23 p19 protein [26, 31–34]. These dendritic cells are also known to produce IL-23 in a TNF-*α*-dependent manner [35].

The above observations suggest that acanthosis present in psoriasis is caused by activation of STAT3 in keratinocytes, which mediates production of IL-22 (Th17/IL-23 axis effector molecule). Although IL12B, IL23R, and IL23A have been identified as psoriasis susceptibility genes, genetic polymorphism has been suggested in the Th17/IL-23 axis that promotes genetic predisposition [36].

Psoriasis improves dramatically after administration of the IL-12/23 p40 monoclonal antibodies ustekinumab and briakinumab [37–40]. Because clinical psoriatic symptoms improve and IL-23 levels decrease after treatment with narrow-band ultraviolet-B (NB-UVB) and TNF-*α* inhibitors, a relationship between excess production of IL-23 and activation of psoriatic disease is indicated [26, 41, 42].

Furthermore, administration of anti-IL-12/23 p40 antibodies results in improvements in psoriasis because they inhibit the Th17/IL-23 axis. However, since TNF-*α* inhibitors also affect production of IL-23 by acting on dermal myeloid dendritic cells, it has been determined that these antibodies inhibit IL-23/Th17 signals [37, 43].

## 4. The Th17/IL-23 Axis in Psoriatic Arthritis

The importance of Th17 cells, IL-23, and Th22 cells in the immunopathology of psoriasis has been previously determined using mice. Can this also be the case for PsA?

Studies involving mouse models are being used to establish the roles of Th17 cells in arthritis, including RA, and as pathogenic cells for many autoimmune and chronic inflammatory diseases. Although T17 cells have been determined to play an important role in pathology of arthritis using mouse models, involvement of Th17 cells and related cytokines in human arthritic disease, including RA, cannot be called definitive. In fact, clinical trials with an anti-IL-17 neutralizing antibody (LY2439821) for RA, with arthritis as the key therapeutic target, are currently being conducted [44]. Despite observing significant improvements, effects are inconclusive when compared to the dramatic results obtained with TNF-*α* inhibitor treatment.

**Figure 1:**
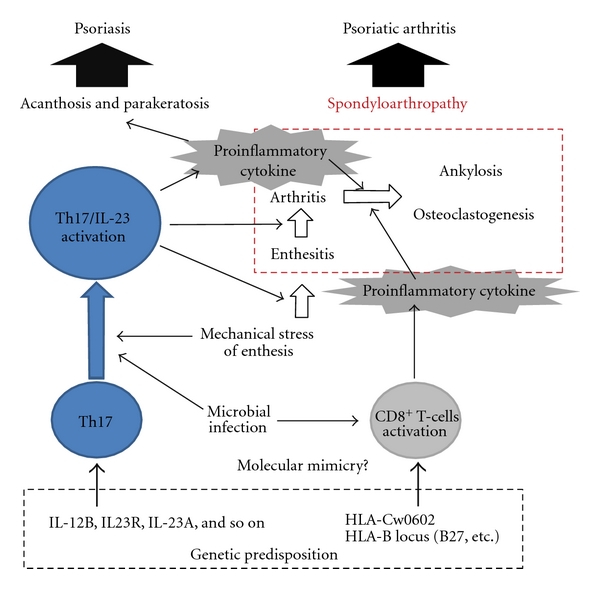
Th17/IL-23 axis, environmental factors and psoriatic arthritis. Environmental factors, such as microbial infections, Koebner phenomenon, trigger Th17/IL-23 and CD8+ T cells activation. Pro-inflammatory cytokines secreted by activated T cells promote inflammation and induce acanthosis, parakeratosis, and spondyloarthropathy.

Similar to dermal cutaneous psoriatic eruptions, significant infiltration of the inflamed synovial membrane by activated CD4+ T cells is observed in PsA [45]. Fibroblasts and T cells in synovial fluid in PsA induce osteoclastogenesis and bone resorption, leading to destructive arthritis via their interactions with receptor activators of nuclear factor *κ*B (RANK), its ligand (RANKL), TNF-*α*, and IL-7. *In vitro* analysis revealed that IL-23 exhibited osteoclastogenesis inductive activities [2, 46] on IL-17, TNF, and RANKL through an independent mechanism [47]. As stated previously, treatment of psoriasis with IL-12/23 p40 antibodies has been dramatically successful. A phase II clinical trial also found significant improvement in terms of the effects of as an IL-12/23 p40 antibody (ustekinumab) on PsA [48]. This suggests the pathological roles of IL-23 in potentiation of osteoclastogenesis and bone erosion in PsA-affected joints. Although immune responses mediated by IL-17 and IL-23 are not as apparent as with TNF-*α*, Th17 cells appear to play an important role in PsA. The immunopathology of PsA/psoriasis, including the Th17/IL/23 axis, is shown in Figure 1.

## 5. Characteristics of the Immunopathology of PsA Synovitis

We have mainly discussed the immunopathological differences between inflammation in cutaneous psoriatic eruptions and synovitis. Organs exhibit differences in their inflammatory reactions; however, this alone cannot explain the precise pathology of PsA. Another useful approach is confirmation of the key differences between RA (the main pathology of which is arthritis) and PsA. At present, RA can be treated using various biopharmaceuticals and pathology of RA is better elucidated than that of PsA. Confirmation of the key differences between PsA and RA synovitis will be extremely useful for developing new treatment strategies for PsA.

PsA synovitis is indicated by hyperplasia of the synovial lining cells and mononuclear cell infiltration, similar to the pathological changes observed in RA [45]. Like RA, ectopic lymphoid neogenesis occurs in the inflamed synovial lining in PsA, and there are peripheral lymph node addressin-positive high endothelial venules (HEV, PNA d  + HEV) accompanied by the expression of the chemokines CXC chemokine ligand (CXCL) 13 and CC chemokine ligand (CCL) 21. Microanatomical bases for germinal center formation are known to exist in PsA [49].

Pathologically, therefore, no characteristic findings are apparent when compared to other types of synovitis, although there are key differences between RA and PsA synovitis. In PsA, the lining layer is relatively thinner than in RA, the sublining layer shows significant infiltration of both T and B cells, and it is characterized by vascular proliferation and proliferation of synovial lining cells.

Cytokines in the synovial fluid have also been compared, and Th1 cytokines (TNF-*α*, IL-1*β*, and IL-10) were found at higher levels in PsA than in RA synovial lining samples. Proinflammatory cytokines, such as IFN-*γ*, IL-12, IL-15, IL-17, and IL-18, also exhibited a higher level of expression in PsA synovial tissue samples. Fibroblasts from the skin and joints of patients with PsA are highly proliferative and secrete the cytokines IL-6, IL-1, and platelet-derived growth factor (PDGF) [50–53].

A study by Kruithof et al. compared the immunological and pathological features of synovitis in spondyloarthropathy (SPA), which includes both RA and PsA [54]. They found that the tissue in this disease was highly vascularized with an increased number of neutrophils and CD163-positive macrophages. In contrast, thickening of the lining layer and an increased number of CD83-positive dendritic cells were more often seen in RA. Thus, it appears that the mechanisms causing synovitis differ between these two diseases.

Clinically, onset of PsA has been determined to occur as enthesitis. Its pathology is considered to be more similar to that of SPA than to RA [2]. In a previous study [54], intracellular staining of tissue samples revealed intracellular citrullinated proteins in 44% of patients with RA and major histocompatibility complex (MHC) human cartilage gp39 peptide complexes (MHC-HC gp39) in 46%. These were not observed in samples from patients with PsA or SPA. Thus, in addition to its clinical picture, PsA is also immunologically and pathologically more similar to SPA than RA. As we described in the previous chapter, Th17/IL-23 axis plays an important role in immune pathogenesis of psoriasis and PsA. This also corresponds to the fact that there are greater increases in the number of peripheral blood Th17 cells in SPA than in RA [55].

Clinically, enthesitis observed in other types of SPA is present in PsA, and it has been predicted that inflammation of the synovial lining adjacent to the entheses will subsequently occur in rapid succession. The synovio-entheseal complex (SEC) is therefore conceptualized as the primary target tissue in PsA [56]. Although nail lesions often accompany PsA, McGonagle et al. considered that the nail matrix could be included in a broad definition, that is, enthesitis of distal interphalangeal (DIP) joints, because although the fibrous tissue continuing from the DIP extensor tendon did not attach to the bone, it continued from the nail matrix [57]. Fibers anchored to the nail plate adhere to the synovial lining and penetrate the nail bed. Nail lesions occur as a result of this enthesitis, indicating the presence of DIP joint lesions.

## 6. Microbial Infections and Onset of Psoriasis and PsA

Disease susceptibility genes in the HLA-B locus, such as HLA-B27, are common in SPA, such as reactive arthritis and ankylosing spondylitis. The HLA-B susceptibility genes HLA-B38, HLA-B39, HLA-B27, and MICA (major-histocompatibility-complex-class-I-related chain A) have been reported to be associated with PsA [3]. Onset of SPA is known to be triggered by microbial infection. Here we outline the factors responsible for PsA and psoriasis caused by microbial infections and activation of natural immunity.

Many studies have investigated the relationship among microbial infections, psoriasis, and PsA. In psoriasis, which is the basis for PsA, *Candida* levels in the salivary gland, alimentary canal, and feces of patients are higher than those in healthy subjects [58, 59]. These *Candida* antigens may lead to psoriatic lesions. We previously stated that an immune reaction via the Th17/IL-23 axis plays an important role in psoriatic lesions and that *Candida albicans* (*Candida* infection) can induce production of Th17 cells, These reactions are heavily dependent upon C-type lectin receptors, such as Dectin-1 and Dectin-2 [60–63], and are indispensable for inducing Th17 cells.

Extrinsic trigger factors are also important in PsA. Bacteria and viruses are particularly important microorganisms. As is the case with psoriasis, *β*-hemolytic group A *Streptococci* are initial causes of onset of PsA. However, in comparison to psoriasis, PsA is rarely triggered by streptococci and is nonspecific [64].

Prinz indicated that in psoriasis and PsA, psoriatic pathology may be triggered by streptococcal antigens and molecular mimicry of epidermal autoantigens [65]. As the superantigens from *Staphylococcus aureus*, *Streptococci*, and *Candida* can all stimulate T-cells, it is possible that T cell stimulation by superantigens is responsible for pathology of psoriasis.

## 7. Activation of Natural Immunity and Psoriasis/PsA

Although medicines rarely trigger these diseases, IFN-*α* is a well-known trigger [66]. Furthermore, psoriatic exacerbation and formation of new cutaneous eruptions have been reported to be caused by administration of IFN-*α* during the treatment of hepatitis C. Additionally, imiquimod, an analog of Toll-like receptor (TLR) 7, while not directly exhibiting antiproliferative activity on viruses, can act on TLRs expressed by monocytes and dendritic cells to promote production of cytokines, such as IFN-*α*, TNF-*α*, and IL-12. It chiefly inhibits viral proliferation via IFN-*α* action. Cutaneous psoriatic eruptions can be induced at external imiquimod sites. This mechanism is considered to involve infiltration of numerous plasmacytoid dendritic cells (pDCs) in the dermis [67]. Activated pDCs produce large amounts of IFN-*α*, which then stimulates myeloid dendritic cells to activate autoimmune T-cell subsets, such as Th17 and Th1, thus inducing psoriatic lesions.

The cutaneous-derived antimicrobial peptide LL-37 also causes psoriatic lesions through the same mechanism. It has been reported that LL37-mediated TLR9 signaling after aggregation with autogenous DNA and activation of pDCs resulted in the onset of psoriasis [68]. Greater production of LL-37 than in the psoriatic epidermis causes the completion of a positive feedback loop, after which the IL-23/Th17 system is activated. This suggests a link between natural immunity and acquired immunity that causes onset of psoriatic.

The types of psoriatic pathology exacerbation caused by natural immunity do not necessarily constitute evidence for PsA exacerbation. However, trauma has been reported to promote PsA [69]. PsA is an arthropathy in which early onset of enthesitis is often present. It has been hypothesized that traumatic stimulation (Koebner phenomenon) of enthesis activates natural immunity mediated by TLRs, which initiates arthropathy. The details of this internal Koebner phenomenon remain unclear, but involvement of cutaneous psoriatic lesions, increased substance P in the synovial lining, and neuropeptides has been indicated [70]. Microbial psoriasis and activation of natural immunity induce Th17 and Th1 cells, which exacerbates autoimmune pathology. This mechanism is an important phenomenon that indicates a relationship between natural immunity and acquired immunity in autoimmune diseases, and we hope that this will be further elucidated in the future.

## 8. CD8+ T Cells and PsA

There is an interesting report regarding the relationship of PsA with microbial infection that is suggestive of PsA pathology. The number of CD4+ T cells in HIV infection is considerably reduced. Thus, in systemic autoimmune diseases, such as RA and systemic lupus erythematosus (SLE) in which CD4+ T cells play an important role, reduced number of CD4+ T cells due to HIV infection can decrease disease activity and direct a patient toward improvement. This reduction in cell number is unfortunately not seen in PsA [71].

This suggests that pathology of PsA differs substantially from that of RA and SLE. It has been hypothesized that in PsA, activation of CD8+ T cells caused by activation of natural immunity is as important as cytokine- and microbial-derived activation. CD4+ and as CD8+ T cells are considered to play important roles in the skin and joints in PsA [72, 73].

This is also suggested by the results of genetic analyses for causative genes of both psoriasis and PsA, such as genome-wide association studies (GWAS). These results showed that HLA-Cw0602 was a disease-susceptibility gene with one of the highest levels of sensitivity [3]. HLA-B38, HLA-B39, HLA-B27, and MICA are known to be important in onset of PsA and are more specific for PsA than for psoriasis. In both diseases, however, these genes are found at the HLA-B locus, which suggests that CD8+ T cells are pathologically important.

Although it has not yet been determined how CD8+ T cells are involved in PsA, they appear to be mainly, or partially, involved in potentiating production of cytokine in the synovial lining and skin. The potentiation of inflammatory cytokines, such as IL-1*β*, IL-2, IL-10, IFN-*γ*, and TNF-*α*, has been observed in the synovial fluid of patients with PsA. These cytokines induce fibroblast proliferation in the synovial lining, thus promoting fibrosis [52, 53, 72], that probably contribute to joint stiffness and ankylosis.

Furthermore, osteoclastogenesis has also been suggested to be potentiated. This would affect bone turnover when these proinflammatory cytokines activate T cells, and upregulated expression of the osteoprotegerin ligand [74].

## 9. Conclusion

Recent advances in immunology have clarified the pathological roles of a newly identified subset of helper T cells, Th17 cells, and IL-23 in autoimmune diseases. Among numerous autoimmune diseases, these discoveries have contributed most to the elucidation of pathology of psoriasis and PsA.

PsA is immunologically more similar to SPA than to RA. It is triggered by microbial infections, activation of natural immunity, and functional mechanical stress at the site of enthesis. Like psoriasis, acquired immunity involving the Th17/IL-23 axis is considered to play an important role in PsA. However, differences have been suggested between arthritis-specific pathology and cutaneous psoriatic lesions.

There have also been advances in analyses of human disease-susceptibility genes, including GWAS and development of targeted therapy using biopharmaceuticals. These results indicate that elucidation of pathology of human immune diseases can now move past simple accounts of immunological phenomena and verification involving mouse models. Accordingly, pathology of human diseases can now be verified by human-based science.

In addition to the evaluation of joints, elucidation of pathology of psoriasis is progressing more rapidly than for arthritis since cutaneous lesions from clinical specimens can be easily collected and therapeutic responses can be easily observed. Thus, although PsA is a rare disease, comparisons and understanding cutaneous inflammation and pathology of PsA have made it possible to make discoveries that would be difficult to detect with joint-based investigations alone. This has contributed not only to elucidation of pathology of PsA but also to that of other autoimmune arthritis. In addition to advances in immunology, feedback, GWAS, and treatment effects of biopharmaceuticals are believed to help in elucidation of pathology of PsA in humans.
